# Commercial DURAClone panels for extending the repertoire of multicolour immunophenotypic panels in an academic flow cytometry laboratory in South Africa

**DOI:** 10.4102/ajlm.v11i1.1720

**Published:** 2022-11-29

**Authors:** Leanne Swart, Melanie Pretorius, Denise Lawrie, Deborah K. Glencross

**Affiliations:** 1 Department of Molecular Medicine and Haematology, Charlotte Maxeke Johannesburg Academic Hospital, National Health Laboratory Service, Johannesburg, South Africa; 2 Department of Molecular Medicine and Haematology, Faculty of Health Sciences, University of the Witwatersrand, Johannesburg, South Africa

**Keywords:** flow cytometry, multicolour, immunophenotyping, lymphoma, leukaemia, plasma cell dyscrasia, acute lymphoblastic leukaemia, B-cell lymphoproliferative disorder, lyophilised reagent, fixed panels

## Abstract

**Background:**

Commercial multicolour fixed immunophenotyping panels can improve flow cytometric diagnostic immunophenotyping repertoire.

**Objective:**

This study validated the commercially available, standardised Beckman Coulter lyophilised DURAClone RE panels to discriminate specific haematolymphoid subtypes.

**Methods:**

We compared the diagnostic capability of the DURAClone acute leukaemia B (ALB), chronic leukaemia B (CLB), and plasma cells (PC) panels to the predicate second-line panels in Charlotte Maxeke Johannesburg Academic Hospital, Johannesburg, South Africa, from April to August 2020. Clinical diagnostic concordance between the in-house second-line immunophenotyping (the predicate method) and DURAClone was established. The ALB panels tested for precursor B-cell acute lymphoblastic leukaemia (*n* = 11) or normal bone marrow haematogones (*n* = 9); CLB panels established haematolymphoid subtypes of mature B-cell lymphoproliferative disorders (B-LPD) (*n* = 20), while PC panels detected plasma cell dyscrasias (PCD) (*n* = 17). Flow cytometer setup and data interpretation to discriminate normal and aberrant immunophenotypes were per manufacturer’s instructions.

**Results:**

There was 100% clinical diagnostic concordance between the predicate and the test panels for second-line diagnostic investigation of B-ALL (with additional CD56), mature B-LPD (with additional discernment of CD81, ROR-1, CD79b and CD43) and PCD.

**Conclusion:**

The DURAClone CLB exceeded the predicate second-line performance, offering extended second-line diagnostic discernment of mature B-LPD subtypes and discernment of CD5+ B-LPD from other non-CD5+ (or CD5–) B-LPD; likewise, the PC panels enabled discovery of PCD. While ALB testing offered no additional diagnostic advantage over existing predicate investigation, CD58 did offer additional information to discern haematogones from B-ALL.

## Introduction

Flow cytometry immunophenotypic analysis is a powerful diagnostic and research tool for investigating haemato-lymphoid malignancies, such as leukaemia, lymphoma, and plasma cell dyscrasias (PCD). Recently, multicolour flow cytometric immunophenotyping has become invaluable for improving detection and detailed immunophenotyping during diagnosis and detection of low frequencies of abnormal or aberrant cell populations during minimal residual disease assessment.^1,2^ Aberrant cell phenotypes are distinguished from normal developmental counterparts^2^ and normal background leucocyte populations^3^ by analysing and comparing several simultaneous cell surface and cytoplasmic antigen expression profiles. Multicolour fluorochrome-conjugated antibody panels generally require antibody concentrations and fluorescence intensity optimisation through titration techniques.^4,5^ These assays can be technically challenging and error-prone due to the addition of multiple individual liquid reagents requiring manual pipetting,^4,6^ thus requiring laboratory technicians skilled in titration technique and flow cytometry,^4,7^ including fluorescence-minus-one, colour compensation, and other technical flow cytometry issues.

Commercially available, standardised, lyophilised, fixed and pre-titrated antibody panels,^8,9,10,11,12,13^ like the Beckman Coulter (BC) DURAClone inventory, including the identification of normal and abnormal B progenitor cells (RE ALB),^14^ various chronic B-cell lymphoproliferative disorders including CD5+ chronic lymphocytic leukaemia (RE CLB)^15^ and normal or abnormal plasma cells (RE PC) panels, offer an attractive alternative to the locally developed, in-house multicolour panels described. Commercial multicolour panels also usually offer the advantage of a standardised instrument and analysis protocols; preset software-guided controls are available to establish optimal standardised instrument settings, verified with abnormal and normal stabilised blood material controls.^9^ In addition, these commercial panels are typically fixed panels supplied in a stable, dry (lyophilised) format with predetermined antibody combinations in a single tube^9^; these qualities enhance laboratory efficiency, reduce technical laboratory error and simplify reagent inventory. Some commercial panels also offer some flexibility in marker combinations by providing ‘open’ fluorescence channels^14,15^ so that liquid drop-in markers can be added to the ‘fixed’ panels to accommodate unique and specific diagnostic requirements of the investigating laboratory.

Our laboratory previously established the positive impact on quality, laboratory workflow and diagnostic usefulness of the commercially available, fixed and standardised BC ClearLLab 10C^16^ multicolour panel system (Beckman Coulter, Miami, Florida, United States). Although this method provides excellent coverage to discover most acute leukaemias,^9,16^ the ClearLLab 10C system lacks additional markers to further identify and discern specific haematolymphoid subtypes, especially within the group of mature B-cell lymphoproliferative disorders and PCD. Specifically, although the BC ClearLLab 10C can easily discern normal B-cell precursors or haematogones from similar immunophenotypes present in minimal residual B-cell acute lymphoblastic leukaemia (B-ALL) disease, the CD58 marker is not included. However, it may be helpful to discriminate disease from normal B-cell precursors during minimal B-ALL disease assessment.^1,2^ The ClearLLab 10C system also lacks markers to discern CD5-positive and CD5-negative mature B-cell lymphoproliferative disorders (LPD), using markers such as CD23, CD43, CD79b, CD81, and ROR-1 recommended for classification by Rawstron et al.^17,18^ Investigation of PCD is also limited if investigated only with the ClearLLab 10C system which lacks CD138, an absolute requirement for identifying plasma cells together with CD38^19^. In our laboratory, we have developed several 2–4-colour in-house, second-line immunophenotyping panels to identify the specific haematolymphoid subtypes mentioned, including markers such as CD23 and FMC-7 for mature B-cell LPD and CD138 with CD56 and CD200, to diagnose PCD. These, however, require a separate repertoire of liquid reagents and necessary flow cytometry technical expertise to set up and run the tests. For a busy site, such as our unit, which processes over 400 samples per month, standardised commercially available panels that can supplement second-line immunophenotypic investigation after ClearLLab10C investigation could dramatically increase the repertoire of markers tested and also improve workflow efficiency in our site.

Existing predicate second-line investigation utilises locally established, in-house, non-standardised and manually assembled liquid fluorochrome-conjugated antibody panels. The primary aim of this study was to evaluate the fixed and standardised, pre-titrated commercially available DURAClone RE ALB, RE CLB and RE PC panels (Beckman Coulter, Mumbai, India) as a comprehensive and compact alternative to our existing second-line immunophenotypic investigation of haematological neoplasms. There were two parts to this study. First, we compared the DURAClone CLB and PC tubes to the laboratory’s in-house predicate 2–4-colour method for the following markers: combinations of CD10, CD23, FMC7 and CD22 to discern mature B-cell lymphoproliferative disorders, or CD19, CD38, CD56, CD200 and CD138 to characterise plasma cells. Secondly, we verified manufacturer-described expression for those additional markers that were not included in the older predicate 2–4-colour method, such as verifying the expression of CD43, CD79b, CD81 and ROR-1 in the CLB tube and CD38 and CD138 with CD27, CD81 and CD56 included in the DURAClone PC tube against expected expression in normal leucocyte population counterparts. We also validated the overall diagnostic outcome of the ALB tube; here, we asked whether the CD58 included in the ALB tubes offered any additional diagnostic advantage in identifying a B-ALL that had been identified in the first-line ClearLLab 10C investigation.

## Methods

### Ethical considerations

The University of the Witwatersrand Health Research Ethics Committee approved the study (Ethics Clearance number M1704129). The study’s objective was to validate the commercially available Beckman Coulter DURAClone PC, CLB and PC fixed-tube, pre-titrated panels as an alternative second-line diagnostic workup to our existing in-house second-line immunophenotypic investigation. The interpretation of flow cytometric data and consequent clinical diagnostic outcomes of the existing in-house, second-line, non-standardised panels were compared to the overall clinical diagnostic outcomes of the DURAClone analyses.

### Study design and site

This study was a prospective observational cohort study conducted according to the Standards for reporting Diagnostics Accuracy.^20^ The work was undertaken from April 2020 to August 2020 and conducted in the National Health Laboratory Service flow cytometry laboratory at the Charlotte Maxeke Johannesburg Academic Hospital in Johannesburg, South Africa. This laboratory performs routine immunophenotypic investigation of leukaemias and lymphomas for both children and adults, referred from Charlotte Maxeke Johannesburg Academic Hospital and other hospitals within the University of the Witwatersrand service precinct, as well as from other centres around South Africa. Morphological assessment of peripheral blood, bone marrow and trephine samples (from the same patients whose samples are tested in the flow cytometry unit) is performed in the sister haematology laboratory; auxiliary molecular and cytogenetic investigations are also performed on site. The laboratory participates in the United Kingdom National External Quality Assurance Scheme (UK NEQAS, Sheffield, United Kingdom) Leukaemia Immunophenotyping Part 1 and Part 2 (interpretation) proficiency testing scheme.^21^

### Testing approach

Immunophenotypic testing of all samples at the Charlotte Maxeke Johannesburg Academic Hospital flow cytometry unit is two-tiered. Firstly, an initial first-line workup is performed with BC ClearLLab 10-C (Beckman Coulter, Miami, Florida, United States). The first workup identifies most acute myeloid and lymphoid leukaemia subtypes, distinguishes early and mature T-cell or B-cell lymphoproliferative disorders, and hints at the presence of a PCD (if a population of brightly expressing CD38 cells is noted). A limitation of this ClearLLab 10C system is that further immunophenotypic characterisation of mature B-cell lymphoproliferative disorders^17^ or PCD^19,22^ requires second-line testing using markers that are not included in the first-line testing. For this study, in-house second-line testing specifically assessed CD23 and FMC-7 expression on mature B-cells or CD38 and CD138 expression on plasma cells. Specifically, in the context of B-LPD and in comparison to our in-house second-line investigation that included CD23 and FMC-7, could DURAClone CLB testing with ROR-1, CD20, CD43, CD79b, and 81 replace CD23 and FMC-7 in discerning CD5 B-cell chronic lymphocytic leukaemia (CLL) from other CD5 and non-CD5 expressing B-LPD.^17,18^

### Samples

After all routine testing with ClearLLab 10C and in-house second-line testing (Table 1), samples having sufficient remnant prepared-cell-concentrate or at least 1 mL remaining of the whole sample were re-tested using one of the test methods (either DURAClone RE ALB, RE CLB or RE PC). The aim was to collect at least 20 clinical specimens for each validation (estimated to be 60 samples). All peripheral blood and bone marrow samples were collected by attending physicians into ethylenediaminetetraacetic acid vacutainer tubes; the single body fluid sample was not collected into an anticoagulant but submitted in a vacutainer tube without anticoagulant. At the end of the study period, a total of 57 anonymised samples were identified for second-line DURAClone comparison, including peripheral blood (*n* = 13), bone marrow (*n* = 43), and a pleural fluid sample (*n* = 1) collected from paediatric and adult patients. For DURAClone ALB testing, 20 bone marrow aspirate samples were tested. Eleven of these patients had a diagnosis of B-ALL (*n* = 11), while nine had immunophenotypically normal haematogones. One of the nine was a follow-up of a B-ALL with normal haematogones and no evidence of residual disease. Twenty samples diagnosed with a B-cell lymphoproliferative disorder by predicate ClearLLab 10C and in-house second-line were referred for DURAClone CLB testing. The CLB testing set comprised 12 bone marrow aspirate samples, seven peripheral blood samples, and a single pleural fluid sample. Due to the decommissioning of the older FACSCalibur flow cytometer during the period of study (the instrument used to undertake the predicate in-house second-line testing), only 17 samples with a diagnosis of a PCD by ClearLLab 10-C and in-house second-line investigation were tested by DURAClone PC panels.

**TABLE 1:** Possible reagent selection for method comparison according to suspected target population undertaken from April 2020 to August 2020 at an academic pathology service in Johannesburg, South Africa.

Possible target population	First-line analysis: ClearLLab 10C panels (BC, Peenya, India)	Second line analysis: in-house antibody panels	Second line analysis: DURAClone RE panel panels under evaluation (Beckman Coulter, Karnataka, India)
B-cell acute lymphoblastic leukaemia work-up or follow-up	ClearLLab 10C B, T, M1 and M2 panels	N/A	RE ALB CD22 APC added in two cases
Mature B-cell lymphoproliferative disorder work-up	ClearLLab 10C B, T, M1 and M2 panels	FMC7-FITC†/CD10-PE‡/CD45-ECD†/CD22-APC§ and CD23-FITC¶/CD20-PE‡/CD45-ECD†/CD5-APC§	RE CLB
Plasma cell dyscrasia work-upor follow-up	ClearLLab 10C B, T, M1 and M2 panels	CD19-FITC§/CD138-PE†/CD45-ECD†/CD200-APC§ and CD38-FITC¶/CD56-PE‡	RE PC CD117 ECD added in a single case

N/A, not applicable; FITC, fluorescein isothiocyanate; PE, phycoerythrin; ECD, phycoerythrin-Texas Red conjugate; APC, allophycocyanin; CD, cluster of differentiation.

Monoclonal Suppliers:

† , Beckman Coulter, Marseille, France.

‡ , Beckman Coulter Inc, Brea, California.

§ , Becton Dickinson, San Jose, California.

¶ , Dako-Agilent, Santa Clara, California. & Invitrogen-Fisher Scientific Inc, Pittsburgh, Pennsylvania.

### Instruments

The Navios^TM^ (BC, Miami, Florida, United States) and the FACSCalibur flow cytometer (Becton Dickinson Biosciences, San Jose, California, United States) were used during this study. The Navios^TM^ flow cytometer was used to analyse the predicate ClearLLab 10C as well as the test DURAClone panels. Internal quality control on the Navios^TM^ included daily assessment of background contamination, cellular events carryover between tubes, and fluorospheres acquisition to verify the flow cytometer optical alignment and fluidics (Flow-Check Pro, BC, Lismeehan, Ireland). In addition, ClearLLab™ Normal and Abnormal process control cells (BC, Lismeehan, Ireland) were used to verify sample processing, acquisition, and analysis. ClearLLab 10C panel acquisition setup was achieved by applying target values set in the manufacturer manual and using BC FlowSet Pro beads to adjust the voltages that enabled optimal detection and separation of dim and bright antigens. After pilot testing (data not shown), the Navios^TM^ instrument ClearLLab 10C settings were deemed appropriate for the DURAClone multicolour data acquisition and analysis.

The FACSCalibur flow cytometer was used to acquire the predicate in-house second-line 2–4-colour fluorescence panels using CellQuest software (BD, San Jose, California, United States). Quality control performed on the BD FACSCalibur included daily assessment of background contamination, carryover, acquisition, and analysis of manufacturer-recommended 3-colour and APC Calibrite beads (Becton Dickinson Biosciences, San Jose, California, United States). Further acquisition and analysis of Immunotrol^TM^ process control (BC, Brea, California, United States) using four monoclonal antibodies, CD14 FITC, CD13 PE, CD45 PERCP and CD3 APC (all Becton Dickinson Biosciences, San Jose, California, United States), was done.

### Sample preparation

Sample preparation included lysing two or more 0.5 mL sample aliquots (dependent on the initial white cell count) with 14.5 mL isotonic ammonium chloride pH 7.1–7.4 (8.99% NH4Cl, 0.84% NaHCO_3_ and 0.0372% ethylenediaminetetraacetic acid; Merck, Darmstadt, Germany) in a 15 mL conical centrifuge tube for 15 min at room temperature. After incubation, samples were spun at 3000 g for 3 min, the supernatant was decanted, while the pellet was washed four times with 14 mL phosphate-buffered saline at pH 7.3 ± 2 (Oxoid Ltd, Basingstoke, United Kingdom) containing 0.09% sodium azide (NaN_3_) (Merck, Darmstadt, Germany) and 0.2% bovine serum albumin (Biowest, Nuaille, France). Following washing, samples were reconstituted to 0.5 mL with phosphate-buffered saline, and the white cell count was determined. Cells were then diluted (or concentrated) to achieve the recommended cellular concentration of ≤ 10 000 cells/µL for monoclonal antibody incubation. Subsequently, 100 µL of this cell concentrate, which contained approximately 10^6^ cells, was added to the ClearLLab 10C and in-house panels (see Table 1). If the sample was deemed to be suitable for inclusion in the study (i.e. had an established diagnosis by predicate ClearLLab 10C), and if there was sufficient remaining material, 100 µL of the remaining cell concentrate was tested with either DURAClone ALB, CLB or PC panels (Table 1). Details of the markers included in these DURAClone panels are outlined in Table 2. Each DURAClone panel has a ‘spare’ capacity for additional markers per the investigator’s need. In our study, in two cases of precursor B-ALL, the marker CD22 (APC) (BC, Miami, Florida, United States) was added in liquid format to the ALB panel to occupy the free APC channel (see Table 2, DURAClone RE ALB panel) to verify CD22 expression in the context of a B-ALL. In a single case of a suspected PCD, liquid CD117 ECD (BC, Miami, Florida, United States) was added to the DURAClone RE PC tube in the ‘free’ ECD channel to demonstrate expression of CD117 in a case of multiple myeloma. After adding the cell aliquot into panels, and, where applicable, the addition of liquid monoclonal reagent, all samples were incubated at room temperature (average 20 °C – 22 °C) in the dark for 15 min. After incubation, all samples were washed once with phosphate-buffered saline and reconstituted to 0.5 mL.

**TABLE 2:** Possible reagent selection for method comparison according to suspected target population validated at the Charlotte Maxeke Johannesburg Academic Hospital flow cytometry laboratory in Johannesburg, South Africa, April 2020 to August 2020.

Fluorochrome DURAClone	FITC	PE	ECD	PC5.5	PC7	APC	APC-A700	APC-A750	PB	KO
RE ALB panel	CD58	Blank	CD34	CD10	CD19	Blank†	CD38	CD20	Blank	CD45
RE CLB panel	CD81	ROR1	Blank	CD79b	CD19	CD5	Blank	CD43	CD20	CD45
RE PC panel	CD81	CD27	Blank‡	CD19	CD200	CD138	Blank	CD56	CD38	CD45

FITC, fluorescein isothiocyanate; PE, phycoerythrin; ECD, phycoerythrin-Texas Red conjugate; PC5.5, phycoerythrin-cyanine5.5; PC7, phycoerythrin-cyanine7 conjugate; APC, allophycocyanin; APC-700, allophycocyanin-Alexa Fluor700; APC-750, allophycocyanin-Alexa Fluor750; PB, pacific blue; KO, krome orange.

† , In two samples, liquid CD22 APC was added into the ‘blank’ channel;

‡ , In one sample, liquid CD117 ECD was added.

### Data acquisition and interpretation of flow cytometric case data

All samples were acquired on a flow cytometer to acquire raw flow cytometric listmode data. For the predicate in-house panels acquired on the FACSCalibur, 5000 (.fcs data) events were collected. Paint-A-Gate software (Becton Dickinson Biosciences, San Jose, California, United States) was used for analysis of raw FACSCalibur flow cytometric data with a primary gating focus using CD45 and side scatter.

**FIGURE 1:**
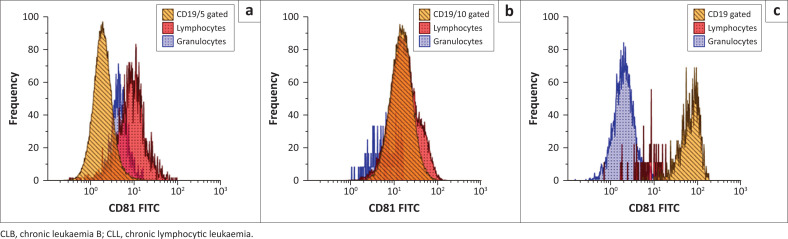
Relative expression of CD81 relative to normal T-cells and granulocytes in the CLB tube validated at the Charlotte Maxeke Johannesburg Academic Hospital flow cytometry laboratory in Johannesburg, South Africa, April 2020 to August 2020. Each plot (a, b and c) shows CD81 expression (*x*-axis) versus frequency (*y*-axis). In A, there is relative (weak) under-expression of CD81 in a patient with CLL gated on CD19+|CD5+; in B, there is relative over-expression of CD81 in a patient with follicular lymphoma gated on CD19+|CD10+ C is a control experiment of a paediatric sample with 20% haematogones showing bright expression of CD81 on normal precursor B-cells gated on weak CD45+| CD19+. Orange represents the B-cells identified with CD19; the blue population represents background granulocytes identified with CD45 and side scatter, and red represents background T-cells gated on CD5 (all markers are included in the CLB panel).

**FIGURE 2:**
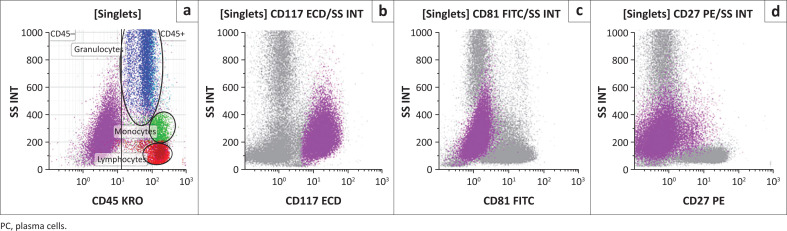
Identification of plasma cells using the DURAClone PC tube validated at the Charlotte Maxeke Johannesburg Academic Hospital flow cytometry laboratory in Johannesburg, South Africa, April 2020 to August 2020. In histogram a, negative CD45 expression of plasma cells is shown (purple), with CD45 positive (background) expression noted amongst granulocytes (blue), monocytes (green) and mature lymphocytes (red). The target plasma cell population were primary-gated on bright CD138 and CD38 to reveal aberrant CD117 expression (b) and under-expression of CD81 (c) and CD27 (d).

For both the ClearLLab 10C and DURAClone panels, at least 50 000 (listmode data) events were acquired on the Navios. Kaluza C™ version 1.1 (BC, Miami, Florida, United States) software was used to analyse all raw listmode data to facilitate clinical interpretation. Kaluza C™ ClearLLab B, T, M1 and M2, and DURAClone panel analysis protocols were developed according to manufacturer specifications.^23^ First, doublets, debris and unlysed red blood cells were excluded, and, where possible, a neoplastic target population was identified in a primary gate using CD45 and side scatter. Thereafter, secondary gating focused on identifying the same target immunophenotype across each of the T, M1 and M2 panels.^9,16^ Finally, simultaneous identification of normal populations in the background was based on local in-house developed gating strategies to identify granulocytes, monocytes and mature lymphocytes using CD45, side scatter characteristics and specific regular expression of the markers studied (Figure 1 and Figure 2).

A similar approach was used for each of the comparative second-line DURAClone RE ALB, RE CLB, and RE PC listmode data analysis but additionally incorporated manufacturer-recommended gating strategies for clinical interpretation of data.^23^ The relative intensity of expression of cell surface antigens was used to discriminate between normal and aberrant immunophenotypes according to manufacturer specifications. Positivity (or no expression) of individual markers was established, and overall haematolymphoid immunophenotypes sub-classified according to the established positive markers outlined in the World Health Organization (WHO) 2016 classification of tumours of haemopoietic and lymphoid tissues.^24^ Specifically, expression of CD27, CD43, CD56, CD58, and CD81, which were not included in the predicate ClearLLab 10C and in-house second-line panel analysis, were verified on background (normal) populations as evidence of a satisfactory internal positive control and proof that the reagents met the manufacturer’s performance specifications. In the ALB analyses, typical secondary backbone markers, including co-expression of CD19/CD10/CD34 enabled further characterisation of markers CD20, CD38, and CD58 and established the presence of normal precursor B-cells (haematogones) or abnormal precursor B-cells. Likewise, for the DURAClone RE CLB, dual expression of either CD19/CD5 or CD19/CD20 was used to identify the ‘target’ or neoplastic B-cell population before specific characterisation of CD43, CD79b, CD81 and ROR1 expression. Plasma cells were defined in the DURAClone RE PC panel by the dual expression of both CD38 and CD138; a normal or malignant plasma cell immunophenotype was subsequently noted following interrogation of markers including CD81, CD27, CD19, CD200, CD56, and CD45.

### Statistical analysis

Clinical diagnostic outcomes were collated into Microsoft Excel (Redmond, Washington, United States) spreadsheets. Marker expressions and specific clinical diagnoses noted for the DURAClone RE ALB, RE CLB, and RE PC panels were compared to the clinical diagnostic outcomes from the existing in-house 2–4-colour antibody panels described (Table 2). The agreement between methods (comparing the final diagnostic outcome) was assessed using contingency tables to determine sensitivity and specificity. True positives were regarded as clinical diagnostic outcomes matching the predicate ClearLLab 10-C with in-house panels, and test system outcomes using ALB, CLB or PC. For example, when a B-cell CLL was diagnosed on the initial ClearLLab investigation with CD23 expression by predicate second-line testing, a diagnosis of a B-cell CLL was confirmed in the CLB tube with positive ROR-1 and CD43 expression. True negatives were those cases where there was no disease noted, either by predicate ClearLLab and second-line testing or by test method with DURAClone panel testing. False positives were defined as those cases where there was no disease noted on predicate ClearLLab with in-house second-line testing, but the disease was noted by respective DURAClone analysis. Likewise, false negatives were defined as disease on predicate first-line ClearLLab and second-line testing, but no disease by respective DURAClone panel testing.

## Results

### Normal and abnormal B progenitor cells panel

Twenty samples were tested with the DURAClone ALB panel. The DURAClone RE ALB evaluation on both routine diagnostic precursor B-ALL samples, and patient samples who were being followed up after therapy for precursor B-ALL, revealed 100% positive and negative agreement to ClearLLab 10C reported outcomes (Table 3). The estimated sensitivity and specificity rates were both 100%. CD58 expression (an additional marker included in the DURAClone ALB tube, but not ClearLLab tubes) enabled further confirmation of the diagnostic outcome reported; here, there was consistent under-expression of CD58 noted on haematogones and consistent over-expression seen on abnormal precursor B-cells (blasts), verifying manufacturer-described CD58 expression. CD22 APC expression (that was added as an additional marker panel in two precursor B-ALL cases to demonstrate that the addition was possible) was verified against the expected disease outcome (positive in two cases of B-ALL) and met the manufacturer’s specifications in the DURAClone RE ALB (Table 3).

**TABLE 3:** Comparison of predicate reagents and DURAClone RE ALB reagent during the assessment of normal and abnormal precursor B-cells undertaken at the Charlotte Maxeke Johannesburg Academic Hospital flow cytometry laboratory in Johannesburg, South Africa, April 2020 to August 2020.

DURAClone RE ALB	ClearLLab 10C and second-line in-house analysis		Total (*N*)
	Disease detected – B-cell acute lymphoblastic leukaemia	No disease – normal haematogones	
Disease detected – B-cell acute lymphoblastic leukaemia	11 (TP)	0 (FP)	11
No disease – normal haematogones	0 (FN)	9 (TN)	9

**Total**	**11 (TP+FN)**	**9 (FP+TN)**	**20**

Note: Percent agreement – Calculation details: Estimated diagnostic sensitivity true positive rate = 100% (TP/(TP+FN)); Estimated diagnostic specificity true negative rate = 100% (TN/(FP+TN)).

TP, true positive; TN, true negative; FP, false positive; FN, false positive.

### Chronic B-cell lymphoproliferative disorder panel

The DURAClone RE CLB panel identified the B-cell target population using a combination of CD19 and CD5 or CD20, as well as surface CD81, ROR1, CD79b, and CD43 expression. Twenty samples were tested with the DURAClone CLB panel. The calculated sensitivity and specificity were 100%, with full diagnostic concordance noted across all cases evaluated (*n* = 20). All CLL cases (*n* = 16/20) showed under-expression (weak) of CD81, CD79b and CD20 (Table 4). Further, surface ROR1 expression was detected in 15 of the 16 (93.75%) CD5-expressing CLL cases; all CLL showed expression of CD43. A single case of follicular lymphoma (*n* = 1) showed a bright expression of CD79b. Three cases (15%) evaluated could not be definitively sub-classified with the in-house second-line panel analysis or the DURAClone RE CLB reagents. An additional control sample (not included in Table 2), with confirmed 20% of B-cell precursors, revealed and confirmed bright CD81 on the normal precursor B-cells (Figure 1).

**TABLE 4:** Comparison of predicate reagents and DURAClone RE CLB reagent during the assessment of mature B-cell lymphoid proliferations undertaken at the Charlotte Maxeke Johannesburg Academic Hospital flow cytometry laboratory in Johannesburg, South Africa, April 2020 to August 2020.

Sample type	Number	Disease/haematologically malignant		
		ClearLLab 10C and second-line in-house diagnostics (diagnosis)	DURAClone RE CLB (diagnosis)	Agreement
Peripheral blood	7	B-cell chronic lymphocytic leukaemia; CD23 positive	B-cell chronic lymphocytic leukaemia; ROR-1 positive	100% diagnostic concordance†
Bone marrow	9	B-cell chronic lymphocytic leukaemia; CD23 positive	B-cell chronic lymphocytic leukaemia; ROR-1 positive	100% diagnostic concordance†
	1	~2% – 3% clonal B-cells; CD19 and CD20 positive with light chain restriction	CD19 and CD20 positive ~3% – 4% aberrant B-cells	100% diagnostic concordance†
	1	Small cell, clonal mature B-cell lymphoproliferative disorder; CD5 negative	Small cell, mature B-cell lymphoproliferative disorder; CD5 negative, CD79b bright	100% diagnostic concordance†
Pleural fluid	1	Follicular lymphoma; CD10 positive	Follicular lymphoma; CD10 and bright CD79b positive	100% diagnostic concordance†

Note: Total – *N* = 20, 100% sensitivity and 100% specificity; all regarded as true positives with full clinical diagnostic concordance.

† , Disease was confirmed across both the predicate ClearLLab 10C with second-line in-house as well as with the index test, DURAClone RE CLB, in all instances.

### Normal and abnormal plasma cells panel

Seventeen samples were tested with the DURAClone PC panel. A plasma cell population was identified by backbone markers CD138 and CD38 in the DURAClone RE PC panel, with subsequent determination of expression of CD19, CD27, CD45, CD56 and CD200. There was cross-panel marker equivalency to the existing in-house panels (Table 5) and clinical outcomes, with 100% agreement achieved, and estimated sensitivity and specificity rates of 100%. In addition, the assessment of CD27 and CD81 on target plasma cells revealed consistent under-expression of CD81 or CD27 (*n* = 14/14) (Figure 2). A single (*n* = 1) PCD had positive surface CD117 expression (Figure 2) on the aberrant identified plasma cell population, in agreement with CD117 expression noted in the matching ClearLLab 10C M2 analysis.

**TABLE 5:** Comparison of predicate reagents and DURAClone RE PC reagent during assessment of normal and abnormal plasma cell populations undertaken at the Charlotte Maxeke Johannesburg Academic Hospital flow cytometry laboratory in Johannesburg, South Africa, April 2020 to August 2020.

Text method: DURAClone RE PC	Predicate method: ClearLLab 10C and second-line in-house analysis		Total
	Disease detected – Plasma cell dyscrasia	No disease – normal or no plasma cells detected	
Disease detected†	15 (TP)	0 (FP)	15
No disease ‡	0 (FN)	2 (TN)	2

**Total (*N*)**	**15 (TP+FN)**	**2 (FP+TN)**	**17**

Note: Percent agreement – Calculation details: Estimated diagnostic sensitivity true positive rate = 100% (TP/(TP+FN)); Estimated diagnostic specificity true negative rate = 100% (TN/(FP+TN)); Diagnostic concordance = 100%.

TP, true positive; TN, true negative; FP, false positive; FN, false positive.

† , Plasma cell dyscrasia – 14 bone marrow aspirate samples with 1% – 45% aberrant plasma cells) (1 peripheral blood sample diagnosed with plasma cell leukaemia, 45% aberrant plasma cells).

‡ , 2 bone marrow aspirate samples with normal plasma cell immunophenotype, less than 1% – 4%.

## Discussion

The recently evaluated,^9,16^ fixed-tube, standardised, pre-titrated reagent system, ClearLLab 10C (including the B-cell, T-cell and M1 and M2 tubes), and ClearLLab LS^11^ were recently implemented to replace our outdated FACSCalibur/Paint-a-gate method for routine immunophenotypic investigation of haematological neoplasms at our laboratory. This new system provides flow cytometric workup for haematological neoplasms and enables processing up to 400 samples a month. Previously, leukaemia flow cytometry testing was limited to simple 2–4-colour analysis; sample setup was manually intensive and error-prone. Systems previously implemented into our laboratory streamlined standardised and automated testing and improved workflow and quality of reporting.^25,26,27^ Similarly, implementing the ClearLLab 10C and LS system in our laboratory^16^ has markedly improved workflow and minimised errors while providing standardised and substantially better quality-controlled sample and data acquisition and comprehensive data. The latter allows for improved target population identification, especially for small populations.

Complementary and supplementary multicolour panel options are published^3,4,10,19,28^ and could theoretically be assembled as required in the lab to provide additional marker panels needed to complete full patient immunophenotypic investigation. However, setting up quality-controlled multicolour panels in low- and middle-income countries is both challenging and less reliable^16^. The ClearLLab system facilitates detailed diagnosis across a broad range of acute leukaemias, and is sufficiently sensitive to detect the presence of a mature B-cell LPD, but it lacks specificity to discern and diagnose different mature B-cell LPD^9^ or confirm PCDs. For example, although the B-cell panel is useful to differentiate precursor B-cell from more mature B-cell LPD, the panel does not accommodate sufficient markers to discern different types of mature LPDs, including CD23, CD43, CD79b and FMC-7 markers necessary to distinguish B-cell LPD per the WHO classification.^24^ Likewise, markers, such as CD58, for diagnosis for subsequent follow-up of precursor B-ALL, or CD79a and CD81, would be of value in the B-cell panel to discern neoplasm from haematogones. Plasma cell dyscrasias are also not easily identified in the ClearLLab system, which lacks CD138 and other markers useful for diagnoses and follow-up of PCD.^22,29,30^ Although CD38, CD56, and CD45, used to identify plasma cells, are included in the ClearLLab panels, plasma cell identification is not definitive. Specifically, CD56, included in the T-cell panel, is assessed separately from CD38 (included in the B-cell and M2 panels). A pre-titrated-monoclonal panel including all three markers would suitably enhance the ClearLLab system; the DURAClone panels used in this study provided this supplement format.

### Normal and abnormal B progenitor cells panel

The expression of CD10, CD19, CD34, CD38, CD20 and CD45 in the DURAClone RE ALB panel showed excellent cross-panel marker expression equivalency and concordant clinical diagnostic outcome when compared to ClearLLab 10C B-cell panel expression. CD58 together with CD38, CD10, CD19, CD34, CD20 and CD45 has potential for sensitive minimal residual disease assessment^31,32^ along with CD81 expression.^31^ CD58 in the index test ALB tube proved to be an excellent adjunct for assessing precursor B-cells and was helpful in discerning abnormal precursor B-cells from normal precursor B-cells (haematogones)^31^. Distinct discordant CD123 and CD34 expression patterns have also been described on haematogones and can be used in a strategy to determine B-ALL minimal residual disease.^32^ CD123 was not however included in the ALB tube. In our predicate method, the ClearLLab 10C system, co-expression of CD123 with CD34 and CD19 assessment within the ClearLLab M2 panel, used to identify precursor B-cells, together with CD19, CD10 and CD34 co-expression in the ClearLLab B-cell tube, therefore offers more diagnostic information than the DURAClone ALB tube alone for the follow-up of precursor B-ALL and residual disease assessment. The ClearLLab 10C system was developed to diagnose early and later B-cell lymphoproliferative disorders; these 10-colour panels are fixed. Thus, additional markers cannot be tested for in the panels. However, additional markers can be added to the RE ALB, fine-tuned toward detecting early B-cell precursors only. CD22 and CD123 could be valuable additions to the ALB panel. For example, a liquid reagent drop-in of CD22-APC in the free APC fluorescence channel of the DURAClone RE ALB panel could provide a baseline assessment of CD22 expression on B-ALL blasts, which would be useful for deciding on anti-CD22 targeted therapy in B-ALL patients. CD123 could also be added in a second available channel to enable further discrimination of disease (B-ALL) from normal haematogones (see Table 2).

### Chronic B-cell lymphoproliferative disorder panel

The expression of CD5, CD19, CD20 and CD45 in the DURAClone RE CLB reagent showed excellent cross-panel marker expression, equivalent to ClearLLab 10C B panel expression, with 100% (*n* = 20/20) and clinical diagnostic outcomes agreement. The additional markers in the DURAClone RE CLB panel, including CD81, ROR1, CD79b and CD43 which are not included in the laboratory predicate ClearLLab system, specifically the B-cell tube, met the requirements for intended use and the manufacturer performance specifications. The DURAClone RE CLB panel proved to be an excellent fixed supplementary panel to replace our outdated second-line investigation for the workup of mature B-cell lymphoid proliferations and proved to be especially useful for discerning CD19/CD5 positive haematolymphoid disease subtypes. The surface CD81, CD79b and CD20 showed consistent under-expression in CLL, while the presence of ROR1 was also helpful in discerning CLL over mantle cell lymphoma as described in previous studies,^17,18,33^ especially when interpreted in conjunction with CD200 expression in the ClearLLab B-cell panel.^34,35^ In the event of a CD19/CD10 co-expressing mature B-cell lymphoid proliferation, CD43 in the DURAClone RE CLB panel was shown to be a useful cell surface marker to discriminate follicular lymphoma from a high-grade B-cell lymphoma. Similarly, CD79b within the DURAClone RE CLB panel, read together with CD38 expression in the ClearLLab B-cell tube, identifies a mature B-cell lymphoid proliferation with plasmacytoid differentiation. As for the ALB panel, free channels in the panel allow for additional flexibility to investigate related markers that are regarded as useful to discern mature B-cell haematolymphoid subtypes. A liquid reagent drop-in of CD23-ECD in the DURAClone RE CLB panel could further confirm B-cell CLL and discern B-cell CLL from other CD19/CD5 co-expressing target populations.^17,18,33^

### Normal and abnormal plasma cells panel

The CD38, CD138, CD45, CD19, CD56, and CD200 expressions in the DURAClone RE PC panel showed excellent cross-panel marker expression and equivalent diagnostic outcome and FACSCalibur/PAG analysis outcomes, with 100% (*n* = 17/17) clinical diagnostic concordance (agreement). In addition, as previously described,^29^ CD27 and CD81^30^ proved to be valuable markers to discern aberrant plasma cells over normal plasma cells. Finally, the usefulness of the RE PC panel can be further extended by adding CD117-ECD^30^ reagents. For one sample, we added CD117-ECD reagents to the PC tube, confirming the presence of malignant plasma cells and minimal residual disease assessment.^14,30.^

### Limitations

Firstly, the sample size per panel evaluated is small, and further studies are needed to confirm the outcomes reported here. Secondly, the fluorochromes used in the panels are specifically designed for use on a BC Navios instrument. Therefore, the products could be used on alternative instruments only if the respective filter setups^23^ (of the particular laboratory’s flow cytometer) can accommodate data collection from the fluorochromes used in the ClearLLab 10C or DURAClone panels.

### Conclusion

This study confirms that the BC DURAClone ALB and CLB panels are suitable to provide additional second-line immunophenotypic workup of precursor B-ALL and mature B-cell lymphoproliferative disorders resepectively, at disease presentation and are suitable to supplement first-line ClearLLab 10C laboratory predicate method testing. The under-expression of CD81, CD79b, and positive CD43 and ROR1 expression, noted in the DURAClone CLB panel specifically assists in distinguishing B-cell CLL from mantle cell lymphoma and other CD5 negative B-cell LPDs.

The DURAClone RE PC panel is suitable for the second-line investigation of PCD. CD27, CD56, CD81 and CD200, together with CD19, CD38 and CD138 in a single analysis, were efficient in identifying aberrant plasma cells.

Although there was diagnostic and individual marker concordance, we did not find the ALB more useful for diagnosing B-ALL over our existing ClearLLab system (utilising the B-cell and M2 tubes). The inclusion of CD58^31^ in the RE ALB tube may however be a valuable adjunct for discerning normal reactive B-cell precursors from the minimal residual disease at follow-up.

Lastly, the potential benefit of using commercialised multicolour lyophilised fixed panel preparations having stable, standardised antibody reagents reduces the risks of technical error, improves laboratory efficiency, and simplifies reagent inventory. In addition, the DURAClone ‘free fluorescence channels’ provide some additional flexibility for a laboratory to use their preferred markers to establish diseases (or not) of their choice.
